# Pre-stroke warfarin enhancement of collateralization in acute ischemic stroke: a retrospective study

**DOI:** 10.1186/s12883-018-1200-7

**Published:** 2018-11-29

**Authors:** Jiaying Zhu, Mengmeng Ma, Yijia Guo, Muke Zhou, Jian Guo, Li He

**Affiliations:** 1 Department of Neurology, West China Hospital, Sichuan University, Chengdu, China; 2 Department of Emergency, Gui Zhou provincial People’s Hospital, Guiyang, China

**Keywords:** Warfarin, Acute ischemic stroke, Collateral circulation

## Abstract

**Background:**

Warfarin therapies not only are used to prevent stroke in patients with high risk of cardioembolism such as patients with atrial fibrillation (AF) and rheumatic heart disease (RHD), but also was associated with lower stroke severity and more favorable functional outcomes in patients with acute ischemic stroke due to middle cerebral artery occlusion. It was speculated that pre-stroke warfarin may promote collateralization and result in reduced stroke severity. This study aimed to investigate the association between pre-stroke warfarin use and leptomeningeal collaterals in patients with acute ischemic stroke due to occlusion of the middle cerebral artery.

**Methods:**

We enrolled consecutive acute ischemic stroke patients (occlusion of the middle cerebral artery within 24 h) with known history of AF and/or RHD at the neurology department of the West China Hospital from May 2011 to April 2017. Computed tomography angiography (CTA) before treatment was used to detect the thrombus. Regional leptomeningeal collateral (rLMC) score based on CTA images was used to assess collateral circulation. Prior use of warfarin was recorded. Univariate and multivariate analyses were performed to detect the association of prior warfarin use with the collateral circulation.

**Results:**

A total of 120 patients were included; 29 (24.2%) were taking warfarin before stroke. The international normalized ratio (INR) in patients with prior warfarin use was 1.53 ± 1.00, compared with 1.02 ± 0.09 in patients without prior warfarin use (*P* < 0.001). Prior oral warfarin therapy was inversely associated with poor rLMC (OR = 0.07, 95%CI 0.01–0.44, *P* = 0.005). There were no associations between prior warfarin use and initial stroke severity or functional outcomes at 3 months.

**Conclusion:**

Warfarin use seems improve collateralization in patients with acute stroke. However, clinical controlled studies should be used to verify this claim.

## Background

Leptomeningeal collaterals are pre-existing anastomoses of distal regions of intracerebral small blood vessels [1, 2]. Collaterals provide the brain tissue with supplementary blood flow in the case of occlusion of primary intracranial arteries to protect brain tissue against irreversible damage [2]. Patients with good leptomeningeal collaterals usually have lower severity of stroke, better response to therapy, and better clinical outcome in acute ischemic stroke [3–6]. Collateral blood vessels could be a therapeutic target in acute ischemic stroke [2]. However, preexisting collaterals depend on cardiac and pulmonary illnesses, metabolic syndrome, hyperuricemia, aging, and genetic factors [2, 4, 7, 8]. Although there are no pharmacologic treatments proven to promote cerebral collateralization currently, some conventional stroke treatments may be available to enhance the collateral circulation [6]. Statins, which have modest antithrombotic pleiotropic effects, have been shown to be associated with the presence of better collaterals in patients with acute stroke [9].

In addition, numerous studies have demonstrated that oral anticoagulants (such as warfarin) reduced the risk of ischemic stroke [10]. Warfarin was also associated with lower stroke severity and more favorable functional outcomes when stroke occurs [11–13]. Although the mechanism for pre-stroke warfarin lowering stroke severity and improving functional outcomes was not very clear, two hypothesizes have been suggested. First, pre-stroke warfarin use may prevent thrombus formation and reduce the size of thrombi. Smaller thrombi cause distal infarctions and lower stroke severity and a more favorable clinical outcome [14, 15]. Secondly, pre-stroke warfarin use may promote collateralization since that the patients who used warfarin before stroke had lower stroke severity comparing with not using warfarin even though the patients of two groups had the similar thrombi and arterial occlusion site [16, 17]. Then it was speculated that pre-stroke warfarin may promote collateralization and result in reduced stroke severity. However, whether the association exit between pre-stroke warfarin use and collaterals still needs to be clarified. In this study, we aimed to evaluate the association between pre-stroke warfarin use and collaterals in acute ischemic stroke patients presenting with occlusion of the middle cerebral artery.

## Methods

### Study population

We performed a retrospective analysis of demographic, clinical, and radiographic data in consecutive patients at the Neurology Department of West China Hospital between May 2011 and April 2017. The inclusion criteria were: (1) ≥18 years of age; (2) history of AF and/or RHD; (3) available computed tomography angiography (CTA) images within 24 h of symptom onset; and (4) CTA detection of acute infarction due to occlusion of the M1 or M2 middle cerebral artery (MCA), with or without occlusion of the internal carotid artery (ICA). The exclusion criteria were: (1) pre-stroke modified Rankin Scale (mRS) score > 2; (2) severe extracranial vascular stenosis detected by CTA; or (3) < 90 days of follow-up.

### Admission CT

All patients underwent non-contrast computed tomography (NCCT) and CTA. Imaging was conducted on a 128-slice Siemens SOMATOM Definition Flash double source CT scanner. NCCT helical scans were performed form the skull base to the vertex using the following parameters: 70 kV, 150 mA, and 5-mm collimation. CTA was performed using: 42 ml of contrast at 6 ml/s, 3 to 5 s delay from injection to scanning, 70 kV, 150 mA, 0.5 s/rotation, and 1.25-mm thick slice. The CTA scan covered the carotid bifurcation to the vertex. Maximum-intensity projection images (20 mm) were reconstructed in the axial, sagittal, and coronal planes.

### Leptomeningeal collateral flow assessment

We assessed the degree of leptomeningeal collateral flow using the regional leptomeningeal collateral (rLMC) score (20 points) [3]. The rLMC score is based on scoring the pial and lenticulostriate arteries in six MCA regions (M1-M6) and in the anterior cerebral artery region and the basal ganglia (0 = no; 1 = less; 2 = equal or more prominent compared with a matching region in the pial artery of the contralateral hemisphere). The arteries in the Sylvian sulcus are given a higher score: 0 = not seen; 2 = less; 4 = same or more prominent compared with the opposite Sylvian sulcus. An rLMC score of 0–10 was defined as poor collateral flow, 11–16 was defined as moderate collateral flow, and 17–20 was defined as good collateral flow [3]. The assessment was performed by four radiologists with extensive experience in acute stroke imaging. The average score was used as the final score for the analyses.

### Data collection

Clinical variables (age, gender, stroke risk factors, admission examinations, previous medications, thrombolytic therapy, and hemorrhagic transformation) were collected for each patient from the stroke database. All biochemical indexes were measured within 24 h of admission. The patient was considered to be using warfarin if they had been using it for at least 3 months prior to stroke and were still using warfarin when stroke occurred. Neurological severity was assessed by trained neurologists using the National Institutes of Health Stroke Scale (NIHSS, < 5 was defined as mild stroke, 5–14 as moderate stroke, and > 14 as severe stroke) [11, 18]. Intracerebral hemorrhage (ICH) was diagnosed according to the European Cooperative Acute Stroke Study (ECASS) criteria. All patients were managed optimally according to the current guidelines, physicians’ experience, and patients’ comorbidities. Follow-up mRS at 3-month after discharge was used to assess the functional outcome. All data collectors were blind to the baseline collateral status.

### Statistical analysis

Statistical analysis was performed using SPSS 22.0 for Windows (IBM, Armonk, NY, USA). The patients were grouped as warfarin users vs. non-warfarin users. The potential determinants of collaterals evaluated based on previous reports in the literature were age, blood pressure, total cholesterol (treated as continuous variables), high-density lipoprotein cholesterol (HDL-C), and low-density lipoprotein cholesterol (LDL-C), and history of stroke, TIA, hypertension, diabetes, hypercholesterolemia, and pre-stroke statin use (treated as dichotomized variables). [19, 20] Continuous variables were expressed as means ± standard deviation or medians (interquartile range) and were analyzed using the Student’s t test or the Mann-Whitney U test between two groups. When comparing percent distributions, the chi-square test was used but Fisher’s exact test was used if the expected number in a category was < 5. Univariate logistic regression was used to determine the association between potential determinants and leptomeningeal collaterals and multivariate logistic regression was used to determine the association between pre-stroke warfarin use and collaterals. Model 1 was adjusted for age, gender, onset to door time; model 2 included model 1 and was additionally adjusted for stroke risk factors; model 3 included model 2 and was additionally adjusted for admission examinations, thrombus location, NIH stroke scale scores; model 4 included model 3 and was additionally adjusted for prior medications. Logistic regression was used to determine the clinical outcomes associated with pre-stroke warfarin use. *P* value < 0.05 was considered statistically significant.

## Results

### Baseline characteristics of the patients

A total of 120 participants were included in the study. Of them, 37 (30.8%) patients were male. Table 1 provided the characteristics of patients with pre-stroke warfarin use or not. Pre-stroke warfarin use was recorded in 29 (24.2%) patients. Compared to patients without warfarin use, those with warfarin use were more likely to be younger age (*P* < 0.001) and to have a higher international normalized ratio (INR) (P < 0.001).

**Table 1:** Clinical characteristics of all participants (*n* = 120)

	All (n = 120)	Pre-stroke warfarin users		P value
		Yes (*n* = 29)	No (*n* = 91)	
Demographics, n (%)				
Age > 60 years	89 (74.2)	12 (41.4)	77 (84.6)	< 0.001
Male	37 (30.8)	8 (27.6)	29 (31.9)	0.664
Onset to door time < 6 h	80 (66.7)	17 (58.6)	63 (69.2)	0.291
Risk factors, n (%)				
Hypertension	60 (50.0)	10 (34.5)	50 (54.9)	0.055
Diabetes	16 (13.3)	3 (10.3)	13 (14.3)	0.759
Atrial fibrillation	112 (93.3)	22 (75.9)	90 (98.9)	< 0.001
Coronary heart disease	17 (14.2)	3 (10.3)	14 (15.4)	0.76
Previous stroke	16 (13.3)	2 (6.9)	14 (15.4)	0.352
Admission examinations, mean ± SD				
INR	1.15 ± 0.54	1.53 ± 1.00	1.02 ± 0.09	< 0.001
Systolic blood pressure	136.88 ± 20.08	133.86 ± 23.74	137.84 ± 18.81	0.356
Diastolic blood pressure	81.31 ± 15.03	82.93 ± 13.70	80.79 ± 15.47	0.507
Serum glucose	7.85 ± 2.40	7.45 ± 2.71	7.97 ± 2.29	0.311
Triglyceride	1.53 ± 1.60	1.90 ± 2.65	1.40 ± 1.08	0.72
Cholesterol	3.97 ± 0.99	3.91 ± 1.29	3.98 ± 0.88	0.325
HDL-C	1.38 ± 0.56	1.36 ± 0.79	1.39 ± 0.47	0.26
LDL-C	2.25 ± 0.72	2.16 ± 0.65	2.29 ± 0.74	0.402
Clinical factors, n (%)				
Thrombus location on CTA				0.078
ICA + M1	28 (23.3)	6 (20.7)	22 (24.2)	
M1	54 (45.0)	9 (31.0)	45 (49.5)	
M2	38 (31.7)	14 (48.3)	24 (26.3)	
NIHSS scores	13 (7–18)	12 (8–16)	13 (7–18)	
Thrombolysis	22 (18.3)	4 (13.8)	18 (19.8)	0.468
Intracranial hemorrhage	27 (22.5)	5 (17.2)	22 (24.2)	0.436
Pre-stroke medications, n (%)				
Antihypertensive drugs	49 (40.8)	10 (34.5)	39 (42.9)	0.424
Antidiabetics	14 (11.7)	3 (10.3)	11 (12.1)	0.549
Antiplatelet drugs	21 (17.5)	2 (6.90)	19 (20.9)	0.084
Statins	21 (17.5)	7 (24.1)	14 (15.4)	0.28

*INR* international normalized ratio, *HDL* high density lipoprotein, *LDL* high density lipoprotein, *CTA* computer tomography angiography;

*ICA* internal carotid artery, *NIHSS* National Institutes of Health Stroke Scale

### Assessment of clinical factors associated with collateral status

Table 2 shows the association between clinical variables and rLMC scores (categorized as poor, moderate, and good). Previous coronary heart disease (*P* = 0.023), low-density lipoprotein cholesterol (*P* = 0.008), NIHSS (P < 0.001), thrombus location (P < 0.001), statin use (*P* = 0.068), and warfarin use (*P* = 0.045) were potential clinical factors associated with collateral flow. In the multivariate models (Table 3), prior warfarin use was inversely associated with poor rLMC (OR = 0.07, 95%CI 0.01–0.44, *P* = 0.005) after adjustment for confounding factors.

**Table 2:** Associated factors with collateral circulation in acute ischemic stroke patients

	Poor (*n* = 43)	Moderate (*n* = 52)	Good (*n* = 25)	P value	Univariate logistic analysis	
					Poor vs. not poor rLMC	Good vs. not good rLMC
Age > 60 years	30 (69.8)	40 (76.9)	15 (60.0)	0.269	0.85 (0.36–1.97)	0.54 (0.21–1.38)
Hypertension history	24 (55.8)	25 (48.1)	11 (44.0)	0.601	1.36 (0.61–3.07)	0.85 (0.33–2.21)
Diabetes	6 (13.9)	8 (15.4)	2 (8.0)	0.664	0.89 (0.28–2.80)	0.48 (0.09–2.44)
Atrial fibrillation	42 (97.7)	48 (92.3)	22 (88.0)	0.282	3.50 (0.38–32.55)	0.61 (0.13–2.97)
Coronary heart disease	9 (20.9)	8 (15.4)	0 (0.0)	0.023	1.46 (0.51–4.17)	–
Previous stroke	5 (11.6)	10 (19.2)	1 (4.00)	0.168	0.55 (0.17–1.76)	0.18 (0.02–1.45)
Systolic blood pressure	34.98 ± 19.98	137.77 ± 21.91	38.28 ± 16.43	0.74	0.99 (0.95–1.02)	1.02 (0.97–1.07)
Diastolic blood pressure	82.67 ± 16.53	80.54 ± 14.04	80.56 ± 14.78	0.761	1.00 (0.96–1.05)	0.93 (0.87–0.99)
Serum glucose	8.33 ± 2.63	7.44 ± 2.17	7.84 ± 2.41	0.252	1.12 (0.98–1.40)	1.09 (0.88–1.34)
Triglyceride	1.51 ± 1.40	1.48 ± 1.99	1.63 ± 0.95	0.106	1.01 (0.69–1.47)	1.06 (0.79–1.39)
Cholesterol	3.92 ± 0.84	3.79 ± 1.09	4.43 ± 0.86	0.173	1.18 (0.75–1.85)	1.83 (1.16–3.40)
HDL-C	1.36 ± 0.44	1.38 ± 0.64	1.44 ± 0.57	0.864	0.91 (0.43–1.94)	1.17 (0.52–2.63)
LDL-C	2.35 ± 0.74	2.04 ± 0.68	2.54 ± 0.67	0.008	1.33 (0.79–2.24)	1.99 (1.07–3.72)
Antihypertensive drugs	17 (39.5)	22 (42.3)	10 (40.0)	0.959	0.89 (0.39–2.03)	0.91 (0.34–2.40)
Antidiabetics	6 (13.9)	7 (13.5)	1 (4.00)	0.44	1.04 (0.32–3.37)	0.27 (0.03–2.31)
Antiplatelet drugs	5 (11.6)	11 (21.2)	5 (20.0)	0.449	0.49 (0.16–1.54)	0.93 (0.29–3.05)
Statins	3 (6.9)	13 (25.0)	5 (20.0)	0.068	0.33 (0.06–0.85)	1.23 (0.40–3.77)
Warfarin	5 (11.6)	15 (28.9)	9 (36.0)	0.045	0.21 (0.10–0.83)	2.11 (0.81–5.48)

*HDL* high density lipoprotein, *LDL* high density lipoprotein, *CTA* computer tomography angiography, *ICA* internal carotid artery, *NIHSS* National Institutes of Health Stroke Scale, *LMC* leptomeningeal collateral

**Table 3:** Association between pre-stroke warfarin use and collaterals

	Poor rLMC vs. Not poor rLMC		Good rLMC vs. Not good rLMC	
	OR (95% CI)	P value	OR (95% CI)	P value
Unadjusted	0.21 (0.10–0.83)	0.021	2.11 (0.81–5.84)	0.125
Model 1	0.18 (0.05–0.61)	0.006	1.92 (0.66–5.62)	0.234
Model 2	0.17 (0.05–0.65)	0.009	2.12 (0.61–7.39)	0.239
Model 3	0.06 (0.01–0.37)	0.002	6.03 (0.94–38.71)	0.058
Model 4	0.07 (0.01–0.44)	0.005	4.80 (0.68–33.79)	0.115

Model 1: Adjusted for age, gender, onset to door time;

Model 2: Adjusted model 1 plus stroke risk factors;

Model 3: Adjusted model 2 plus admission examinations, thrombus location, NIH stroke scale scores;

Model 4: Adjusted model 3 plus prior medications

### Association of Prior Warfarin use with stroke severity, Thrombus location, and clinical outcomes

Table 4 shows the association between prior warfarin use and initial stroke severity, thrombus location, and functional outcome. No significant difference was observed between prior warfarin use and stroke severity or follow-up mRS. Compared with patients without warfarin use exposure, thrombus location (M2 vs. ICA + M1/M1) was significantly different in warfarin users (OR = 0.38, 95%CI 0.16–0.91, *P* = 0.03). After adjustment for age, gender, previous antiplatelet and statins, warfarin users had a tendency to have distal vessel occlusion (OR = 0.37, 95%CI 0.13–1.03, *P* = 0.056).

**Table 4:** Association between pre-stroke warfarin use and clinical outcomes

	Univariate analysis		Multivariate analysis	
	OR (95% CI)	P value	OR (95% CI)	P value
NIHSS scores				
> 14 vs. 0–14	0.74 (0.31–1.76)	0.487	–	–
> 5 vs. 0–5	1.18 (0.39–3.53)	0.762	–	–
Thrombus location				
ICA + M1 vs. M1/M2	0.82 (0.29–2.27)	0.699	–	–
ICA + M1/M1 vs. M2	0.38 (0.16–0.91)	0.03	0.37 (0.13–1.03)	0.056*
Follow-up mRS scores				
0–3 vs. 4–6	1.43 (0.59–3.41)	0.425	–	–
0–2 vs. 3–6	1.49 (0.65–3.46)	0.348	–	–

*Adjusted age, gender, previous antiplatelet and statins, *NIHSS* National Institutes of Health Stroke Scale

*ICA* internal carotid artery, *mRS* modified Rankin Scale

## Discussion

We performed a retrospective analysis of demographic, clinical, and radiographic data in consecutive patients with acute ischemic stroke presenting with the occlusion of middle cerebral artery to evaluate the association between pre-stroke warfarin use and collaterals by CTA images [21]. We found that pre-stroke oral warfarin use was inversely associated with poor leptomeningeal collaterals as identified. It showed that pre-stroke warfarin use may improve the poor cerebral collateral grade among acute ischemic stroke patients presenting with a major arterial occlusion by CTA images which is in accord with the hypothesis [10, 13].

This result also demonstrates a previously unidentified mechanism by which warfarin could lower the risks of poor collateral and reduce stroke severity. It was shown when intracranial artery occludes, secondary microthrombus may form in the distal small blood vessels of occluded artery [10, 15]. This will interfere with collateral circulations in the small vessels. Consequently, warfarin may enhance or facilitate collaterals by 1) preventing the secondary microthrombus formation in the distal small blood vessels of occluded artery, 2) promoting the lysis of thrombi in the distal small vessels and speeding up the resolution process of these thrombi at stroke onset.

The international normalized ratio (INR) value has confirmed an inverse association between admission INR and infarct size in acute ischemic stroke [11, 22–24]. In patients with higher INR (eg, an INR of 2–3), thrombus formation will be prevented and the resolution process of the new thrombus will be accelerated [25]. Indeed, one study conducted in Japan demonstrated that INR controlled between 1.60 and 1.99 is not effective for reducing the severity of ischemic stroke in patients with non-valvular atrial fibrillation [22]. Another study performed in the United States showed that therapeutic anticoagulation (INR ≥2) was associated with lower odds of moderate or severe stroke [11]. In this study, the INR values of the patients with pre-stroke warfarin were relatively lower. This may explain the result of this study that collateral enhancement was only in the patients with poor rLMC compared with other patients. Therefore, it may be needed to explore the underlying effect of the INR on collaterals and stroke severity in the future.

Previous studies have confirmed that vitamin K antagonists, such as warfarin, or non-vitamin antagonist oral anticoagulants (NOACs) reduce the risk of ischemic stroke [26]. Additionally, the current guidelines recommend warfarin or NOACs for stroke prevention in high-risk patients with AF or RHD. Nevertheless, many patients still fail to receive proper stroke prevention in the real world. A previous study showed that only 61.8% outpatients with AF (CHADS2 score ≥ 2) were treated with warfarin or NOACs [27]. In this study, we found that only 29 (31.9%) patients were treated with warfarin, and therefore the treatment rate is far below the rates observed in western countries. In addition, current guidelines suggest that a target INR between 2.0 to 3.0 is considered as effective [28]. Because of the high risk of intracranial hemorrhage in the Asian population, Japanese guidelines recommend that patients > 70 years of age should be controlled with a target INR of 1.60–2.60 [29]. In the United States, a large, nationwide and registry study indicated that in patients with acute ischemic stroke and a known history of AF, 30% were receiving some form of oral antithrombotic therapy before stroke onset, but that 64% of the warfarin-treated patients were receiving sub-therapeutic doses [11].

The presence of collateral cerebral circulation has been shown to be associated with better functional outcomes after stroke [3–6]. After acute ischemic stroke onset, collateral vessels provide the brain tissue with supplementary blood flow. Guidelines are available regarding the methods clinically available or under trial to promote collateral circulation after stroke (head position, induced hypertension, volume expansion, external counter pulsation, bypass surgery, albumin, nitric oxide, TNF-α inhibitors, statins [6]); but these methods are examined in the context of stroke management, not prevention. Nevertheless, some factors are known to be associated with collaterals. Previous studies have shown negative associations between hypertension and collaterals, and positive associations between statin use and collaterals [5, 9]. In addition, age, ischemic preconditioning, and cardiovascular risk factors are considered to be associated with collateral circulation patency [3, 30, 31]. Particularly, metabolic syndrome, high blood uric acid levels, and age have been associated with poor collaterals [4]. In the present study, the multivariable analysis showed that pre-stroke oral warfarin use was associated with collaterals, independently of age, gender, onset to door time, stroke risk factors, admission examinations, thrombus location, NIH stroke scale scores, and pre-stroke medications (model 4). This may be one reason why pre-stroke warfarin use affects stroke severity and clinical outcome. Nevertheless, additional studies in a larger population are necessary to corroborate our observations.

The major challenge for antithrombotic therapy among patients with AF or RHD is the risk of bleeding. In the present study, 22.5% of the patients with acute ischemic stroke were detected with ICH. A previous report indicated that anticoagulant treatment after stroke onset was associated with significant increase in symptomatic intracranial bleeding [32], but we did not find that pre-stroke warfarin use was associated with ICH, which is supported by a previous study [13]. Nevertheless, due to the relatively small sample size in the present study, interpretation should be made with caution. There are several limitations to our study. First, the sample size was relatively small and we failed to perform subgroup analyses; hence, potential heterogeneity exists in the study population. Secondly, this study did not control for the use of previous antiplatelet therapy. Finally, based on the INR, most patients in the present study were found to receive inadequate antithrombotic therapy.

## Conclusions

The results showed that prior warfarin use was inversely associated with the risk for poor collaterals in acute ischemic stroke patients presenting with occlusion of the middle cerebral artery, suggesting that warfarin use seems improve collateralization in patients with acute stroke. However, clinical controlled studies should be used to verify this claim.

## Abbreviations

AF: Atrial fibrillation
CT: Computer tomography
CTA: Computer tomography angiography
ECASS: European Cooperative Acute Stroke Study
HDL: High density lipoprotein
ICA: Internal carotid artery
ICH: Intracerebral hemorrhage
INR: International normalized ratio
LDL: High density lipoprotein
MCA: Middle cerebral artery
MRI: Magnetic resonance imaging
mRS: Modified Rankin Scale
NCCT: Non-contrast computer tomography
NIHSS: National Institutes of Health Stroke Scale
NOACs: Non–vitamin antagonist oral anticoagulants
OR: Odds ratio
RHD: Rheumatic heart disease
rLMC: Regional leptomeningeal collateral
SD: Standard difference
